# Ubiquitin E3 ligase MARCH7 promotes ovarian tumor growth

**DOI:** 10.18632/oncotarget.3650

**Published:** 2015-03-26

**Authors:** Jianguo Hu, Ying Meng, Tinghe Yu, Lina Hu, Ming Mao

**Affiliations:** ^1^ Department of Obstetrics and Gynecology, Second Affiliated Hospital, Chongqing Medical University, Chongqing, China

**Keywords:** ovarian cancer, MARCH7, NF-Kb, Wnt, β-catenin

## Abstract

Ubiquitin E3 ligase MARCH7 is involved in T cell proliferation and neuronal development. We found that expression of MARCH7 was higher in ovarian cancer tissues than normal ovarian tissues. Silencing MARCH7 decreased cell proliferation, migration, and invasion. Ectopic expression of MARCH7 increased cell proliferation, migration and invasion. Silencing MARCH7 prevented ovarian cancer growth in mice. Silencing MARCH7 inhibited NFkB and Wnt/β-catenin pathway. In agreement, ectopically expressed MARCH7 activated NFkB and Wnt/β-catenin pathways. Finally, MARCH7 was regulated by miR-101. Thus, MARCH7 is oncogenic and a potential target (oncotarget) for ovarian cancer therapy.

## INTRODUCTION

The membrane-associated RING-CH (MARCH) proteins belong to the RING finger protein family of E3 ubiquitin ligases, consisting of 11 members in mammals. MARCH proteins have numerous cellular functions, which include immune regulation, protein quality control, membrane trafficking, and spermatogenesis [1]. MARCH7 is a member of the MARCH family, which consists of approximately 690 amino acids within a single RING finger domain [2]. Previous studies have suggested that MARCH7 plays an important role in T cell proliferation and neuronal development [3]. MARCH7 is expressed in multiple types of cells and tissues, including stem cells and precursor cells [4], suggesting its possible role in cell and tissue differentiation. However, little is known about the cellular localization and function of MARCH7 in ovarian carcinoma.

In this study, we have observed an aberrant expression and localization of MARCH7 in ovarian cancer tissues by immunohistochemical analyses. Additionally, we have elucidated the functions of MARCH7 in ovarian cancer. Our results show that MARCH7 participates in the regulation of cytoskeleton re-organization, cellular migration and invasion, cell proliferation, and tumorigenesis in ovarian carcinoma cells. At the same time, we found that MARCH 7 could modulate nuclear factor kB (NF-kB) and Wnt/β-catenin pathways. Further, we identified MARCH7 was an authentic target of miR-101.

## RESULTS

### Aberrant expression of MARCH7 in ovarian carcinoma tissues

**Figure 1:**
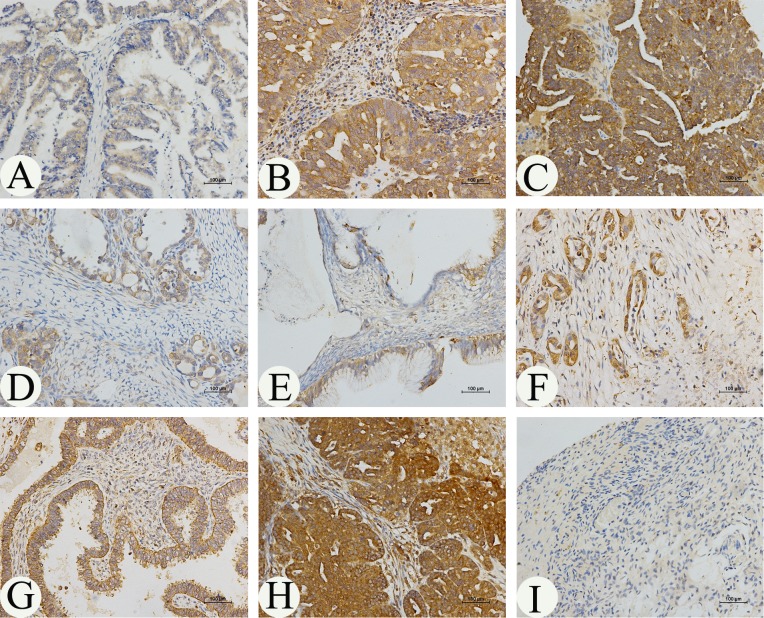
Immunohistochemical analysis of *MARCH7* expression in ovarian cancer MARCH7 was predominantly localized in the (**A**) plasma membrane, (B, C, D, E) cytoplasm. The expression of *MARCH7* in different types of ovarian cancer samples. (**A**). serous papillary adenocarcinoma (stage I); (**B**). serous papillary adenocarcinoma (stage IIIA); (**C**). serous papillary adenocarcinoma (stage IIIC); (**D**). mucinous adenocarcinoma mucinous adenocarcinoma (stage IA); (**E**). mucinous adenocarcinoma mucinous adenocarcinoma (stage IB) ; (**F**). mucinous adenocarcinoma mucinous adenocarcinoma (stage III); (**G**). endometrioid adenocarcinoma(stage I); (**H**). endometrioid adenocarcinoma(stage III); (**I**). normal ovarian tissue. Original magnification, 200X.

The expression profile of MARCH 7 in ovarian cancer is not yet fully elucidated. We examined the expression pattern of MARCH7 in normal ovary and ovarian cancer tissue samples using IHC. The expression of MARCH7 was significantly higher in ovarian carcinoma samples than that in normal ovarian samples (Fig. 1 and Table 1). MARCH7 was predominantly localized on the plasma membrane, and cytoplasm (Fig. 1A-H). MARCH7 expression was significantly higher in epithelial ovarian cancer samples than that in normal ovary tissues (P < 0.05; Table 1). To determine the correlation of MARCH7 expression with cancer type and cancer stage, all cancer samples were grouped into histologic types (serous papillary adenocarcinoma, mucinous adenocarcinoma, and endometrioid adenocarcinoma) (Fig. 1A-H). The differently expression of MARCH7 between serous adenocarcinoma and other histologic type of the tumor was not significant (P>0.05). MARCH7 immunostaining was significantly higher in tumor samples in advanced stages (stage III/IV) as compared to those in the early stages (stage I/II) disease (P < 0.01). Further, the staining intensity significantly correlated with the tumor grade (grades 2–3 versus 1, P < 0.01). However, the associations between MARCH7 expression and age were not significant (P > 0.05; Table 1).

**Table 1:** Association of MARCH7 expression with clinicopathological characteristics in 158 patients of EOC

	No. of patients	MARCH7 expression		P value
	(n=158)	Low no.(%)	High no.(%)	
Characteristics				
Age(years)				>0.5
<50	83	36(43.37%)	47(56.63%)	
≥50	75	35(46.67%)	40(53.33%)	
Normal ovarian	20	18(90%)	2(10%)	<0.05
Cancer tissues	138	79(57.25)	59(42.75%)	
FIGO stage				
I/II	103	74(71.84%)	29(28.16%)	<0.001
III/IV	35	5(14.29%)	30(85.71%)	
Grade				
1	30	23(76.67%)	7(23.33%)	
2	36	24(66.67%)	12(33.33%)	
3	72	30(41.67%)	42(58.33%)	
		Grade 2-3 versus 1		<0.05
Tumor type				
Serous	120	67(32.81%)	53(67.19%)	
Mucinous	14	11(81.25%)	3(18.75%)	
Endometrioid	4	1(75%)	3(25%)	
		Serous versus non-serous		
				>0.005

### MARCH7 expression in ovarian cancer cell lines

**Figure 2:**
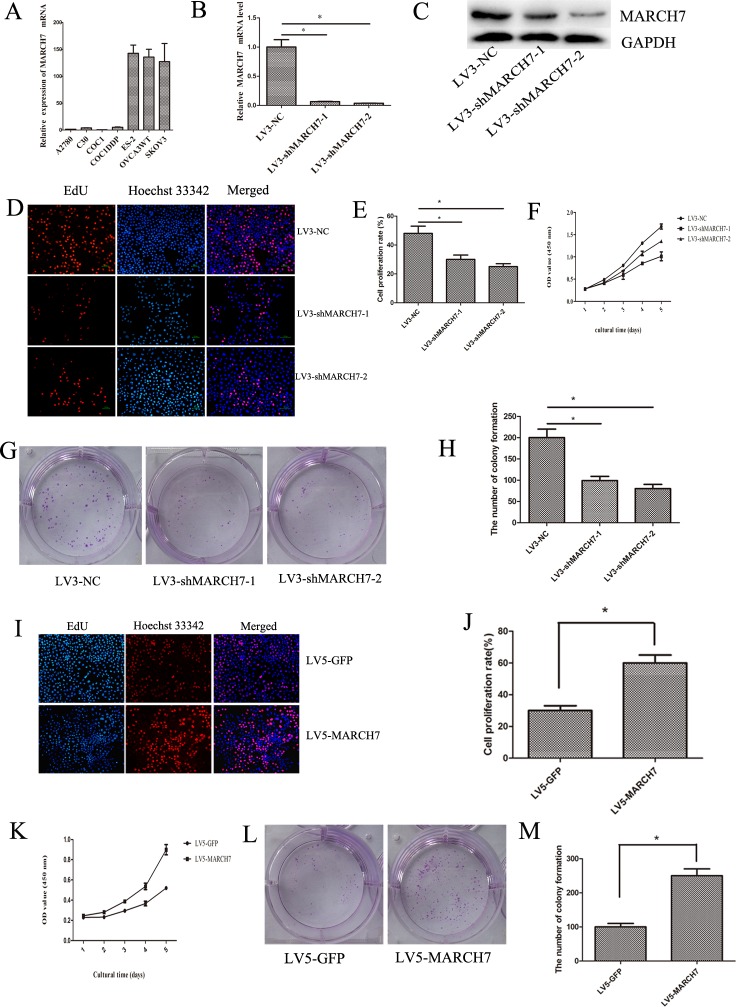
*MARCH7* regulated the proliferation of ovarian cancer SKOV3 and A2780 cells (**A**) The relative expression of MARCH7 mRNA in ovarian cancer cell lines. (**B**, **C**) MARCH7 mRNA and protein level were down-regulated by infected with LV3-shMARCH7-1 or LV3-shMARCH7-2. (**D**, **E**) Ovarian cancer SKOV3 cells were infected with LV3-NC, LV3-shMARCH7-1 and LV3-shMARCH7-2. Cell proliferation was assessed by EdU. The proliferation rate of LV3-shMARCH7-1 and LV3-shMARCH7-2 cells was lower than that of LV3-NC cells. Original magnification, 200X. (**F**) Cell proliferation was determined by CCK-8 assay. (**G**, **H**) Colony formation assay. Skov3 cells infected with Lv3-shMarch7-1 or Lv3-shMarch7-2 were cultured by seeding 1,000 cells in 6-well plates and enumerating the number of colonies formed in 2 weeks. The number of colony formation of LV3-shMARCH7-1 or LV3-shMARCH7-2 was lower as compared with LV3-NC. (**I**, **J**) Ovarian cancer A2780 cells were infected with LV5-GFP or LV5-MARCH7. Then cell proliferation was determined by EdU. Original magnification, 200X. The proliferation rate of LV5-MARCH7 cells was increased compared with LV5-GFP cells. (**K**) Cell proliferation was determined by CCK-8 assay. (**L**, **M**) Colony formation assay. A2780 cells infected with LV5-MARCH7 were cultured by seeding 1000 cells in 6-well plates and counting the number of colonies formed in 2 weeks. The number of colony formation of LV5-MARCH7 was higher as compared with LV5-GFP. Error bars represent standard error. * p < 0.05, and **p < 0.001.

The expression of MARCH7 was investigated in 7 cell lines at the mRNA level by real-time quantitative PCR (qPCR) to select suitable cell lines for functional assays. Of these, MARCH7 expression was higher in the SKOV3, CaOV-3, and Es-2 cell lines, than in the A2780 cell line (Fig. 2A). Therefore, A2780 cell line was selected for exogenous expression; SKOV3 cell was selected for down -regulation of MARCH7 to determine the MARCH7 functions. The mRNA and protein level of MARCH7 was decreased in LV3-shMARCH7-1 or LV3-shMARCH7-2 infected SKOV3 cells compared with LV3-NC SKOV3 cells (Fig. 2B and 2C).

### MARCH7 regulates cellular proliferation

Our data showed that downregulation of MARCH7 using LV3-shMARCH7-1 and LV3-shMARCH7-2 could inhibit cell proliferation in ovarian cancer SKOV3 cell (P < 0.05; Fig. 2D, 2E and 2F). The efficiency of colony formation had decreased in LV3-shMARCH7-1 or LV3-shMARCH7-2 infected SKOV3 cells (P<0.05) (Fig. 2G and 2H).

On the other hand, we investigated the overexpression of MARCH7 in A2780 cell by exposing them to LV5-MARCH7. Our data showed that overexpression of MARCH7 in A2780 cells resulted in an increase in cell proliferation compared to LV-5-GFP-exposed cells (P < 0.05; Fig. 2I, 2J and 2K). This was consistent with the exogenous expression of MARCH7 that increased the colony forming capacity in contrast with LV-5-GFP infected cells (P < 0.05) (Fig. 2L and 2M). However, MARCH7 knockdown did not induce cell apoptosis (data not presented).

### MARCH7 expression modulates cellular migration, invasion *in vitro* and induces F-actin remodeling

**Figure 3:**
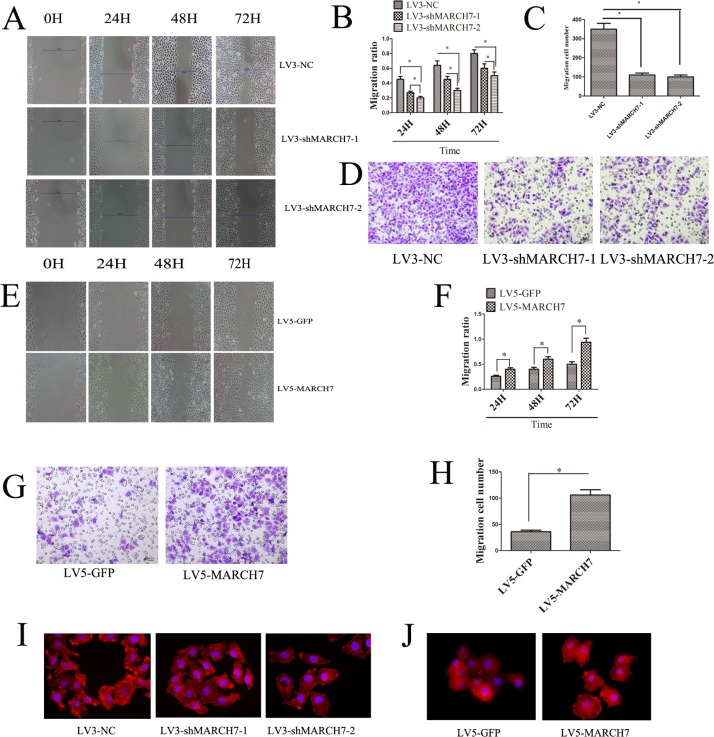
(**A**, **B**) Ovarian cancer SKOV3 cells migration ability was detected by the wound healing assay. The migration of LV3-shMARCH7-1 and LV3-shMARCH7-2 infected SKOV3 cells was lower as compared with LV3-NC infected cells. (**C**, **D**) Ovarian cancer SKOV3 cells invasion ability was detected by Matrigel invasion assays. The invasion ability of LV3-shMARCH7-1 and LV3-shMARCH7-2 infected SKOV3 cells was decreased compared with LV3-NC infected cells. (**E**, **F**) Ovarian cancer A2780 cells migration ability was detected by wound healing assay. The migration ability of LV5-MARCH7 infected A2780 cells was increased compared with LV3-NC infected cells. (**G**, **H**) Ovarian cancer A2780 cells invasion ability was detected by Matrigel invasion assays. The invasion ability of LV5-MARCH7 infected A2780 cells was increased compared with LV5-GFP infected cells. (**I**, **J**). F-actin staining. Original magnification, 400X. Error bars represent standard error. * p < 0.05, and **p < 0.001.

Migration of cells and invasion of tissues are important prerequisites for tumor progression and metastasis. To investigate whether MARCH7 modulated cellular migration and invasion, we performed a matrigel invasion assay and wound healing test. Wound-healing and trans-well invasion assays both demonstrated that the migration and invasion capabilities of SKOV3 cell were significantly suppressed when MARCH7 was silenced by LV3-shMARCH7-1 or LV3-shMARCH7-2 (p < 0.05) (Fig. 3A, 3B, 3C, and 3D). At the same time, we found that the migration and invasion capabilities of A2780 cells were significantly promoted when MARCH7 was overexpressed with a lentiviral vector expressing MARCH7 (LV5-MARCH7) (P < 0.05) (Fig. 3E, 3F, 3G, and 3H). Cellular migration and invasion is dependent on localized actin polymerization at the leading edge of the cells. Polymerization of globular actin leads to the formation of long fibrous molecules, F-actin. In eukaryotic cells, cell migration requires the formation of cell membrane extensions containing actin filaments [5]. Because overexpression of MARCH7 in A2780 cells caused a marked increase in the cellular migration and invasion, and silencing of MARCH7 expression in SK0V3 cells caused a marked decreased in the cellular migration and invasion, we analyzed the alterations in the pattern of the F-actin in SKOV3 and A2780 by silencing or ectopic expression MARCH7 respectively. In LV3-NC infected SKOV3 cells, F-actin staining was predominantly localized in the cellular outgrowth and projections. In contrast, in LV3-shMARCH7-1 or LV3-shMARCH7-2 infected SKOV3 cells, F-actin staining was homogenous throughout the cytoplasm, and the formation of membrane ruffles and lamellipodia was prevented (Fig. 3I). LV5-GFP-infected A2780 cells displayed some small lamellipodia and ruffles. In contrast, LV5-MARCH7 infected A2780 cells showed F-actin reorganization in membrane ruffles and lamellipodia (Fig. 3J). These results suggest that MARCH7 can modulate cellular dynamics by reorganization of the actin cytoskeleton.

### TGF-β1, TNF-α and IL-1β regulate the expression of MARCH7

**Figure 4:**
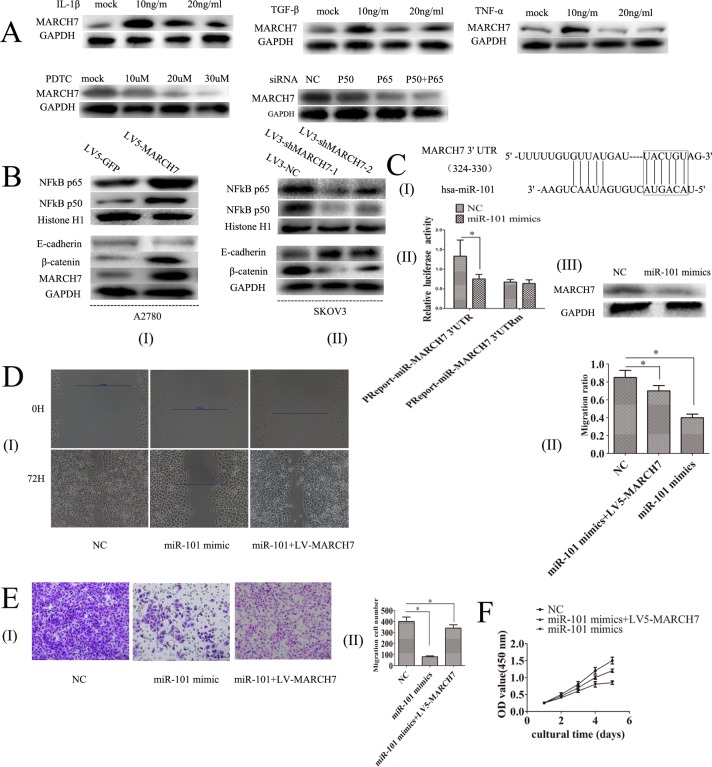
(**A**) The expression of MARCH7 protein level in SKOV3 cells was regulated by TGF-β1, TNF-α, IL-1β, PTDC, NFkB P50 and NFkB P65.(**B**) The protein expression level in SKOV3 and A2780 cells of NFkB P65, NFkB P50, E-cadherin and β-catenin was detected by western blot. (**C**-I) A putative binding site targeted by miR-101 was predicted to be located in 3′UTR of MARCH7 mRNA. (**C**-II)SKOV3 cells were co-transfected with miR-101 mimics or control RNA [negative control (NC)] with luciferase reporter plasmids containing either wild type (pMIR-MARCH7-3UTR) or mutant 3′UTR (pMIR-MARCH7-3UTRm) of MARCH7 gene. Luciferase expression was measured. The fold changes of relative luciferase activity in miR-101 mimics with indicated plasmids transfected cells were normalized to NC with corresponding indicated plasmids transtected cells, respectively. (**C**-III)The expression of MARCH7 protein levels was detected by western blot. (**D**) Ovarian cancer SKOV3 cells migration ability was detected by the wound healing assay. (E) Ovarian cancer SKOV3 cells invasion ability was detected by Matrigel invasion assays. (**F**) Cell proliferation was determined by CCK-8 assay. * p<0.05, and **p<0.001.

Transforming growth factor (TGF)-β1, tumor necrosis factor-alpha (TNF-α) and Interleukin-1β are expressed in ovarian cancer, which can promote ovarian tumorigenesis through an inflammatory response [6-8]. Hence, we explored the possibility of whether TGF-β1, TNF-α, and interleukin-1β can mediate MARCH7 expression in SKOV3 cells. Ovarian cancer SKOV3 cells were treated with TGF-β1 (0, 10, 20, 30 ng/mL), TNF-α (0, 10, 20, 30 ng/mL) or interleukin-1β (0, 10, 20, 30 ng/mL) for 48 hours. TGF-β1 increased the mRNA and protein level of MARCH7 at 10 ng/mL than that untreated. However, the mRNA and protein levels were lower in cells treated with TGF-β1 20 ng/mL or 30 ng/mL. The changes in the mRNA and protein levels of MARCH7 in response to TNF-α or interleukin-1β was similar to that with TGF-β1 treatment at (0, 10, 20, 30 ng/mL) (Fig. 4A and Fig. S1A-C). Our results indicated that TGF-β1, TNF-α, and interleukin-1β regulated MARCH7 expression to promote tumor metastasis of ovarian cancer.

### Ectopic expression of MARCH7 increased NF-κB and Wnt/β-catenin signal pathway luciferase reporter activity

Our data indicated that MARCH7 might have been involved in cellular proliferation, migration, and invasion. Hence, we believe that MARCH7 might mediate some signal pathway, which is associated with ovarian cancer. We detected NF-κB, Notch, P53, STAT3, and Wnt/β-catenin signal pathway luciferase reporter activity by ectopic expression of MARCH7 in ovarian cancer A2780 cells infected with LV5-MARCH7. We found that ectopic expression of MARCH7 in ovarian cancer A2780 cells significantly increased the NF-κB and Wnt/β-catenin pathway luciferase reporter activities (P < 0.01) (Fig. S1D). Hence, we hypothesize that MARCH7 may regulate NF-κB and Wnt/β-catenin signal pathway.

### MARCH7 mediates NF-kB pathway in ovarian cancer SKOV3 and A2780 cells

**Figure 5:**
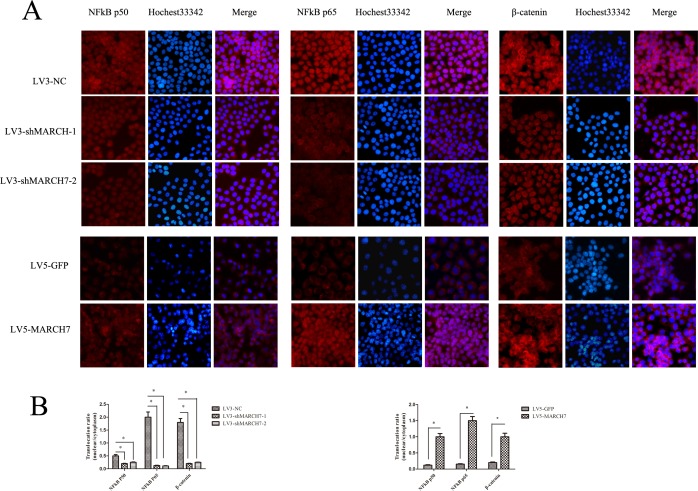
(**A**) The expression of NFkB P65, NFkB P50, and β-catenin was detected by immunofluorescence staining in ovarian cancer SKOV3 cells (LV3-shMARCH7-1 or LV3-shMARCH7-2 infected) and A2780 cells (LV5-MARCH7 infected). (**B**). The nuclear translocation of NFkB P65, NFkB P50, and β-catenin was decreased in LV3-shMARCH7-1 or LV3-shMARCH7-2 infected ovarian cancer SKOV3 cells as compared with LV3-NC infected cells. The nuclear translocation of NFkB P65, NFkB P50, and β-catenin was increased in LV5-MARCH7 infected ovarian cancer A2780 cells compared with LV5-GFP infected cells. Original magnification ×200. Data are expressed as Mean ± SD from three independent experiments. * p < 0.05, and **p < 0.001.

The silencing of MARCH7 in SKOV3 cells caused a marked decrease in NF-κB luciferase activity (P<0.05), that was consistent with the above results (Fig. S1E). P65 and P50 protein levels were decreased in SKOV3 cells when MARCH7 was silenced (Fig. 4B(I)). Ectopic *MARCH7* gene expression in A2780 cells increased the protein level P65 and P50 (Fig. 4B(II)). We tested whether MARCH7 regulated nuclear localization of individual NF-κB in ovarian cancer cells such as SKOV3 and A2780. The decrease in nuclear translocation of NF kB p65 and P50 after transfection to SKOV3 cells were observed with LV3-shMARCH7-1 or LV3-shMARCH7-2 as compared to that of LV3-NC (P<0.05) (Fig. 5A and 5B). Increase in nuclear translocation of NF kB p65 and P50 were observed after infection of A2780 cells with LV5-MARCH7 as compared with that of LV5-GFP (P < 0.05) (Fig. 5A and 5B).

Next, we determined whether NF-κB pathway could regulate MARCH7. Inhibition of NF-κB signaling in SKOV3 cells by PTDC caused a reduction in the levels of mRNA and protein of MARCH7, with the reduction corresponding to an increase in the PTDC concentration (0, 10, 30 50 uM) (Fig. 4A and S1F). The mRNA and protein level of MARCH7 in SKOV3 cells were lowered after transfection with P50 or P65 (Fig. 4A and S1G). Our data suggested MARCH7 interact with NF kB pathway.

### MARCH7 regulates Wnt/β-catenin pathway in ovarian cancer SKOV3 and A2780 cell

Consistent with the above results, the level of β-catenin protein expressed in LV5-MARCH7-infected A2780 cells were significantly higher than that of LV5-GFP-infected A2780 cells (Fig. 4B(I)). In contrast, LV3-shMARCH7-1 or LV3-shMARCH7-2 infected cells decreased TOP-flash luciferase activity, and the expression of β-catenin protein than that of LV3-NC-infected SKOV3 cells (Fig.4B(II) and S1H).

Non-phosphorylated β-catenin has been identified as the transcriptionally active form of β-catenin in the nucleus. Therefore, we determined if MARCH7 promoted β-catenin translocation to the nuclei of SKOV3 and A2780 cells. A2780 cells exposed to LV5-MARCH7 resulted in significantly higher levels of β-catenin proteins in both the cytoplasm and the nuclei, relative to LV5-GFP-infected A2780 cells (P < 0.05) (Fig. 5A and 5B). Conversely, LV3-shMARCH7-1 or LV3-shMARCH7-2 infected SKOV3 cells showed a decrease in both, the cytoplasmic and nuclear expression levels of β-catenin relative to LV3-NC-infected SKOV3 cells (P < 0.05) (Fig. 5A and 5B). These findings suggested that *MARCH7* activation of β-catenin results in nuclear translocation and accumulation, thereby enabling it to regulate expression of genes associated with tumorigenesis.

*C-myc, sp5, lef1* play key roles in the Wnt/β-catenin signaling pathway [9-11]. Therefore, we determined the expression of their respective mRNAs by qPCR by silencing *MARCH7* in SKOV3 cells, and ectopic expression of MARCH7 in A2780 cells. The results showed that mRNA levels of *c-myc, sp5, lef1* were significantly higher in LV5-MARCH7-infected A2780 cells (P < 0.05) (Fig. S1I); whereas, they were significantly lower in LV3-shMARCH7-1 or LV3-shMARCH7-2 infected SKOV3 cells, relative to LV3-NC infected SKOV3 cells (P < 0.05) (Fig. S1J).

### MARCH7 regulates the protein levels of E-cadherin in ovarian cancer SKOV3 cells

Intercellular adhesion correlates with the presence of E-cadherin and catenin complexes. It regulates β-catenin transport to the cell nucleus to activate the transcription of many genes [12, 13]. Therefore, we explored whether MARCH7 regulated the expression level of E-cadherin protein. The results showed that protein levels of E-cadherin were significantly decreased in LV5-MARCH7-infected A2780 cells (Fig. 4B(I)); whereas, they were significantly increased in LV3-shMARCH7-1 or LV3-shMARCH7-2 infected SKOV3 cells, relative to LV3-NC infected SKOV3 cells (Fig. 4B(II))

### MiR-101 regulates MARCH7 expression in ovarian cancers

Bioinformatics analyses using multiple algorithms showed that *MARCH7* is a predictive target of miR-101(http://www.targetscan.org/). Thus, we experimentally verified whether miR-101 modulated MARCH7 expression in SKOV3 cells. We predicted that miR-101-specific binding site was located within the 3′UTR of MARCH7 mRNA (Fig. 4C(I)). We constructed a vector to investigate if miR-101 could directly target *MARCH7 3′UTR*. We found that miR-101 markedly inhibited luciferase activity when MARCH7 3′UTR was inserted downstream of luciferase cDNA in our reporter vector (pMIR-MARCH73UTR). In contrast, no significant suppressive effect on luciferase activity was observed in cells transfected with a control vector with mutant MARCH7 3′UTR (MIR- MARCH73UTRm), when miR-101 expression was elevated (Fig. 4C(II)). We also found that miR-101 mimics could downregulate the mRNA and protein level of MARCH7 (Fig. 4C(III) and S1K).

We also found that expression of miR-101 inhibited proliferation, migration and invasion of SKOV3 cells. The phenotypes can be partially restored expression of a miR-101 resistant MARCH7 (Fig. 4D, 4E and 4F). These data indicate that MARCH7 is a direct target of miR-101.

### Downregulation of MARCH7 abolished tumorigenicity of ovarian cancer SKOV3 cell

**Figure 6:**
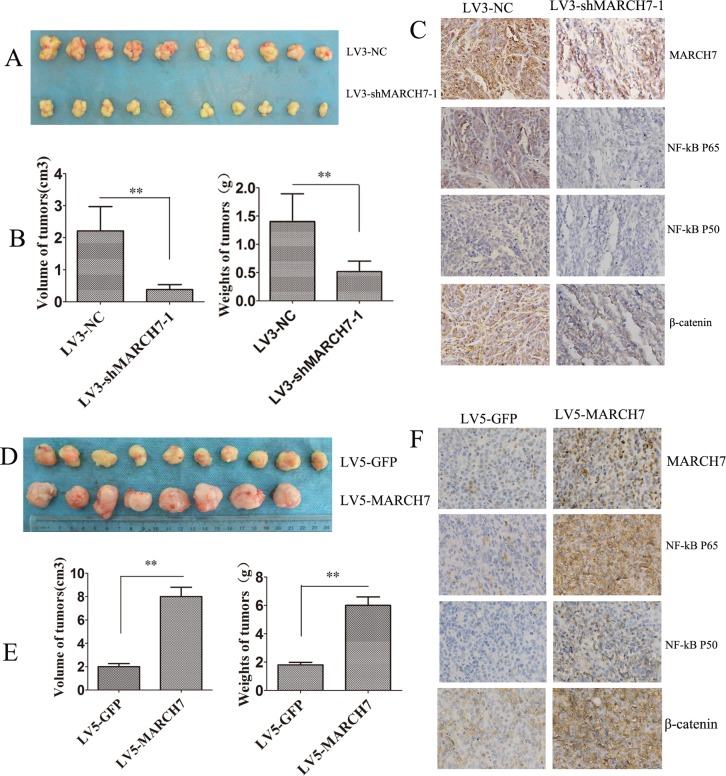
*MARCH7* regulated tumorigenesis in nude mice model (**A**, **B**) Mean tumor volume and weight on day 21 after tumor cell injection. LV3-shMARCH7-1 or LV3-NC infected SKOV3 cells were implanted s.c. into the left armpit. (**C**). Immunohistochemical analysis of MARCH7, NFkB P65, NFkB P50, and β-catenin expression were performed on tumor xenografts. Representative images are shown (original magnification ×200). (**D**, **E**) Mean tumor volume and weight on day 21 after tumor cell injection. LV5-GFP or LV5-MARCH7 infected A2780 cells were implanted s.c. into the left armpit. (**F**) Immunohistochemical analysis of MARCH7, NFkB P65, NFkB P50, and β-catenin expression were performed on tumor xenografts. Representative images are shown (original magnification ×200). * p < 0.05, and **p < 0.001.

The role of MARCH7 in tumor formation of ovarian cancer SKOV3 cells was investigated in an animal model. LV3-shMARCH7-1 and LV3-NC infected SKOV3 cells formed tumors in all nude mice. The average weight of tumors was significantly lower in LV3-shMARCH7-1 infected group than that in LV3-NC infected group (P < 0.01) (Fig. 6A and 6B). The average volume of tumors in LV3-shMARCH7-1 infected group was significantly lower than that of the LV3-NC infected group (P < 0.01; Fig. 6A and 6B). IHC revealed that the expression of MARCH7, P50, P65 and β-catenin in tumors from LV3-shMARCH7-1 infected group was lower than that in the LV3-NC infected group (P < 0.05) (Fig. 6C and S2A). These data show that silencing *MARCH7* blocks tumor formation *in vivo*. *MARCH7* can regulate the expression of P50, P65 and β-catenin *in vivo*. We also found that A2780 cells infected LV5-MARCH7 increased the average weight of tumors and the average volume of tumors than that of the LV5-GFP infected group (P < 0.01; Fig. 6D and 6E). IHC revealed that the expression of MARCH7, P50, P65 and β-catenin in tumors from LV5-MARCH7 infected group was increased than that in the LV5-GFP infected group (P < 0.05) (Fig. 6F and S2B). These data show that *MARCH7* promoted tumor formation *in vivo*.

## DISCUSSION

In this study, we noted that *MARCH7* expression was higher in ovarian cancer tissues, compared to normal ovarian tissues. We also observed that overexpression of *MARCH7* in ovarian cancer correlated with tumor stage and histological grades. Our results showed that up-regulation of *MARCH7* expression in ovarian cancer increased cellular migration, invasion, and cell proliferation in vitro with remarkable changes in the cytoskeleton. These results suggest that elevated levels of *MARCH7* aids in the progress of ovarian cancer, and promotes an aggressive behavior, indicating that *MARCH7* may function as a novel tumor marker and a potential therapeutic target for ovarian cancer. Our data also demonstrates that *MARCH7* can regulate nuclear factor kB (NF-kB) and Wnt/β-catenin pathway, and that MIR-101 controls MARCH7 by targeting MATCH7 3′UTR region.

In addition to the investigation of MARCH7 in ovarian cancer, we explored the role of *MARCH7* in ovarian cancer. We found that inhibiting MARCH7 expression in ovarian cancer SKOV3 cells suppressed cancer cell activities, whereas overexpression *MARCH7* in ovarian cancer A2780 cells increased cancer cell activities such as growth, migration, and invasion. In a living cell, the actin cytoskeleton continuously undergoes remodeling at a constant rate. This dynamics are based on predictive controlled equilibrium, and a dynamic balance of actin filaments. This regulation is crucial for cell motility, and cancer cell invasion [14-16]. In our study, we found that *MARCH7* regulated localization in the cellular, membrane lamellipodia, and ruffles. These results indicate that *MARCH7* is a novel regulator of ovarian cancer progression through its effect on actin cytoskeletal alterations. Most importantly, downregulation of MARCH7 in ovarian cancer cells could abolish their tumorigenecity.

Until now, there is no study implicating *MARCH7* in the pathogenesis of cancer, the mechanisms of *MARCH7* in cancer development and progression are poorly known. NF κB and Wnt/β-catenin pathway are involved in cancer development and progression, in various carcinomas including ovarian cancer, breast cancer, endometrial cancer, cervical cancer [17-23]. In human ovarian carcinoma, there is constitutive activation of NF-κB, which leads to uncontrolled growth, anti-apoptosis, and immune evasion [24]. In this study, we found that ectopic expression of *MARCH7* in ovarian cancer cells increased NF-κB activity. In contrast, downregulation of *MARCH7* in ovarian cancer decreased NF-κB activity, especially the p65 and p50 subunits of NF-kB protein. NF-kB p65 is the major subunit involved in ovarian cancer development and progression [25]. In our experiments, the silencing of *MARCH7* expression in ovarian cancer reduced P50 and P65 protein levels; ectopic expression of *MARCH7* in ovarian cancer increased the protein level of P50 and P65. We also observed that upregulation MARCH7 in ovarian cancer A2780 cells promoted the P50 and P65 translocation to the nucleus; downregulation of *MARCH7* in ovarian cancer SKOV3 cells inhibited P50 and P65 translocation to the nucleus. To further study the relation between MARCH7 and NF kB, we tried inhibiting NF kB with PDTC (Ammonium pyrrolidinedithiocarbamate), P50 siRNA or/and P65 siRNA. We found that PDTC, P50 siRNA or/and P65 siRNA significantly reduced the mRNA and protein level of MARCH7. This suggested that NF kB can regulate the expression of MARCH7 in ovarian cancer. Our data indicated that NF kB interaction with MARCH7 was crucial for the progress of ovarian cancer.

Elevation of β-catenin in the cytosol and the nucleus can occur independently through various pathways [25, 26]. Furthermore, β-catenin is a central factor in canonical Wnt signaling [27]. We found that ectopic expression of MARCH7 in ovarian cancer A2780 cells increased the expression of β-catenin in the cytoplasm and promote its translocation to the nucleus. Downregulation of *MARCH7* in ovarian cancer SKOV3 cells decreased the expression of β-catenin in the cytoplasm and repressed its translocation to the nucleus. These results confirmed that *MARCH7* promoted β-catenin translocation to the nucleus in ovarian cancer cells. Once within the nucleus, β-catenin regulates expression of genes involved in the activation of the Wnt/β-catenin signaling pathway, including sp5, lef1 and c-myc. We had shown that MARCH7 upregulated β-catenin, therefore, to test whether MARCH7 may also be implicated in regulating the Wnt/β-catenin pathway, we investigated its regulatory effect on TopFlash reporter activity, *sp5, lef1* and *c-myc*. Our results showed that *MARCH7* mediated TopFlash reporter activity and expression of *sp5*, *lef1,* and *c-myc* mRNA. Based on these findings, we concluded that *MARCH7* participated in Wnt/β-catenin signaling in human ovarian cancer cells.

β-catenin involved in Wnt/β-catenin pathway and intercellular adhesion, which is causally correlated with the existence of E-cadherin and catenin complexes. The adhesive properties of E-cadherin correlated with the junctions with β-catenin. In cancer cells, it plays an important role in cell signaling due to the Wnt/β-catenin signaling pathway, which impedes β-catenin degradation in the cytoplasm, resulting in protein accumulation and transport to the cell nucleus to activate the transcription of many genes [12, 13]. The results of this study showed that ectopic expression of *MARCH7* in ovarian cancer A2780 cells reduced the protein levels of E-cadherin. In contrast, silencing of *MARCH7* expression in ovarian cancer SKOV3 cells significantly increased the protein level of E-cadherin. It indicates that *MARCH7* reduced E-cadherin to promote β-catenin transport to the cell nucleus.

MicroRNA-101 is decreased in epithelial ovarian cancer compared with normal tissue. MiR-101 ectopic expression in epithelial ovarian cancer cell lines resulted in increased apoptosis, decreased cellular proliferation, invasiveness, and reduced growth of tumor xenografts [28, 29]. Our results showed that miR-101 mimics reduced the mRNA and protein level of MARCH7 in ovarian cancer SKOV3 cells. We further confirmed miR-101 inhibited MARCH7 expression by targeting its mRNA 3′UTR.

In conclusion, this study confirms that MARCH7 is a tumor promoting gene in humans with ovarian cancer, which was involved in NFkB and Wnt/β-catenin pathway (Fig. S2C). This also suggests that MARCH7 may be a potential therapeutic target in patients with epithelial ovarian cancer.

## MATERIALS AND METHODS

### Tissue specimens

The tissue microarray slides containing malignant and benign ovarian tissues (n=158) were obtained from US Biomax Inc cancer tissue bank collection (US Biomax Inc., MD, USA). The Ethics Committee of the Chongqing Medical University approved the study documents and the use of archived cancer tissues. All patients provided a written informed consent.

### Cell culture, transfection procedure, and reagents

SKOV3 human ovarian cancer cell line derived from the ascites from a 64 year old caucasian female with an ovarian tumor. The A2780 cell line was established from tumor tissue from an untreated patient. The ES-2 cell line was established from a surgical tumor specimen taken from a 47 year old black woman. CAOV-3 human ovarian cancer cell line derived from a 54 years old caucasian female with ovarian adenocarcinoma. The COC1 cell line was derived from the ascites of patients with poorly differentiated ovarian cancer. Cisplatin-resistant COC1/DDP, which is derived from its parental ovarian cancer cell line COC1 by stepwise selection *in vitro* using cisplatin. Human ovarian cancer cells were cultured in Rosewall Park Memorial Institute (RPMI) 1640 medium, containing 10% fetal bovine serum and antibiotics, and incubated in an atmosphere with 5% carbon dioxide at 37°C. Double-strand oligonucleotides corresponding to the target sequences were synthesized by Genepharma Co., Ltd. (Shanghai, China). The following sequences were targeted for human MARCH7, NF-Kb p50, and NF-kB P65 small interfering RNA (siRNA), respectively. MARCH7-1:5′-GCACUUGGGAGUAAUUUGA-3′; MARCH7-2:5′-GCACACGUGUCCGAUUUAU-3′; NF-Kb p50: 5′- GATTGAGGAGAAACGTAAA-3′; NF-Kb p65:5′-GTCACTCTAACGTATGCAA-3′ and NC (negative control) siRNA: 5′-UUCUUCGAAGGUGUCACGUTT-3′. Lentiviral vector expressing shRNA targeting MARCH7 (named LV3- shMarch7-1 and LV3-shMarch7-2) and MARCH7-lentiviral expression vector (named LV5-March7) were provided from Genepharma Co., Ltd. (Shanghai, China). miR-101 Mimics (sense: 5′-UACAGUACUGUGAUAACUGAA-3′) were synthesized at Ruibo Biotech (Ruibo Biotechnology, Guangzhou, China).

### Immunohistochemistry

Immunohistochemistry (IHC) was performed according to the SP kit instructions (SP-9000, ZSGB-BIO, Beijing, China). After dewaxing and hydration, the sections were heated in citrate buffer (pH 6.0, Sigma-Aldrich, USA) in a microwave oven for 20 minutes for antigen retrieval. Further, the sections were cooled naturally to room temperature. The sections were washed thrice for 3 minutes per cycle. Subsequently, the sections were incubated in 3% aquae hydrogenii dioxidi for 15 minutes at room temperature, and washed thrice with phosphate buffered saline (PBS) for 3 minutes per cycles. The sections were blocked with 5% donkey serum (ab7475 Abcam Company) for 30 minutes at 37°C. Anti-MARCH7 rabbit polyclonal antibody (1:100, bs-9341R, Bioss, Beijing, China) was incubated with the sections overnight at 4°C. Negative controls included omission of primary antibody and use of irrelevant primary antibodies. The corresponding secondary antibodies that were conjugated to horseradish peroxidase (Bioss Biotechnology) were incubated with the sections for an hour at room temperature. The sections were washed thrice in PBS for 3 minutes per cycle. The sections were incubated in horseradish enzyme-labeled chain avidin solution (Bioss Biotechnology) for 30 minutes at 37°C, and washed in PBS for 3 minutes x3 cycles. The proteins were visualized by diaminobenzidine. All the sections were observed by 3 independent pathologists using a light microscope. The staining data were obtained from manually recorded reports. Staining intensity was graded on a 0–3 scale as follows: 0 (absence of staining), 1 (weakly stained), 2 (moderately stained), and 3 (strongly stained). The percentage of positive tumor cells was scored as follows: 0 (absence of tumor cells), 1 (<33% tumor cells), 2 (33–66% tumor cells) and 3 (>66% tumor cells). Immunohistochemical score (ranging from 0 to 9) was calculated by multiplying the intensity score and the percentage score [30].

### Quantitative real-time polymerase chain reaction (PCR)

Total RNA was isolated using an RNA pure High-purity Total RNA Rapid Extraction Kit (BioTeke, RP1201, China), as per the instructions provided in the kit. cDNA was synthesized using the iSCRIPT cDNA synthesis kit (Bio-Rad). The primers used for amplifying MARCH7, sp5, lef1, c-myc and GAPDH were synthesized by Guangzhou Funeng Co., Ltd. The real-time PCR kit was purchased from Guangzhou Funeng Co., Ltd. PCR conditions were 95°C for 10 seconds, 60°C for 20 seconds, 72°C for 10 seconds. Each sample was analyzed in triplicates. Relative quantification of mRNA was performed using the comparative threshold cycles (CT) method. This value was used to plot the gene expression employing the formula 2^−Δ ΔCT^.

### Detection of protein expression by Western blotting

Expression of NF-kB p50, P65, MARCH7, E-cadherin and beta catenin protein was analyzed by the Western blot method [9]. The primary antibodies used included polyclonal rabbit anti-MARCH7 (1:1000; ab84130; Abcam Inc., Cambridge, MA, USA); polyclonal rabbit anti-NF-kB p65 (1:1000; ab7970; Abcam Inc., Cambridge, MA, USA); polyclonal rabbit anti-NF-kB p50 (1:1000; ab7971; Abcam Inc., Cambridge, MA, USA); anti-beta catenin antibody rabbit polyclonal antibody (1:500, bs-1165R, Bioss, Beijing, China) and polyclonal rabbit anti-GAPDH (1:1000; AB10016; Sangon Biotech, Shanghai, China). The band density was analyzed using a gel imaging system and compared against an internal control.

### Cell proliferation assay

Cell proliferation was determined using the CCK-8 assay as described previously [10], and EdU assay was performed using the Cell-Light TM EdU imaging detecting kit according to the instructions in the kit (Ruibo Biotechnology, Guangzhou, China). EdU is a thymidine analog that can be used to label cells undergoing DNA replication [11].

### Colony formation assay

SKOV3 cells infected with LV3-shMarch7-1 or LV3-shMarch7-2, and A2780 cells infected with LV5-March7 were cultured by seeding 1000 cells in 6-well plates, and the number of colonies formed was checked after 2 weeks. All experiments were triplicated.

### Dual-luciferase reporter gene assay

Luciferase reporter gene assay was performed using the Dual-Luciferase Reporter Assay System (Promega) according to the instructions provided by the manufacturer. For MARCH7 3′ UTR luciferase reporter assay, wild type or mutant reporter constructs (termed WT or Mut; purchased from Genepharma Co., Ltd., Shanghai, China) were co-transfected into skov3 cells in 24-well plates with 100 nM miR-101 or 100 nM miR-NC and Renilla plasmid by using Endofectin™-Plus (GeneCopoeia). TOPflash reporter plasmid and NF kB reporter plasmids were purchased from Shanghai Qcbio Science & Technologies Co., Ltd. (Shanghai, China). Reporter gene assay was performed 48 hours post-transfection using the Dual Luciferase Assay System (Promega, Madison, WI). Firefly luciferase activity was normalized for transfection efficiency using the corresponding Renilla luciferase activity. All experiments were performed at least 3 times.

### Wound healing assay and Matrigel invasion assays

Migration of SKOV3 and A2780 cells were analyzed using the wound-healing assay *in vitro*. Cells were seeded into 6-well plates and cultivated until 90% growth confluence. Wounds were afflicted by scraping the monolayer cells with a sterile pipette tip. At 0, 24, 48 and 72 hours after the wounding, cells were observed under low power in an Olympus light microscope. The distance between the 2 wounds were measured at each time point, and expressed as the average percent of wound closure as compared to that at zero time. Invasion of SKOV3 and A2780 cells were evaluated by Matrigel invasion assays. For Transwell invasion assays, the upper side of an 8 μm pore, 6.5-mm polycarbonate transwell filter (Corning, New York, NY) chamber was uniformly coated with Matrigel basement membrane matrix (BD Biosciences, Bedford, MA) for 2 h at 37°C before the cells were added. A total of 5×10^4^ cells were seeded into the top chamber of a trans-well filter (in triplicate) and incubated for 48 hours. Invasive cells on the lower side of the filter, were fixed in 4% paraformaldehyde, stained in 0.5% crystal violet (Beyotime), and counted using a microscope. A total of 5 fields were counted for each transwell filter. Each field was counted and photographed at 200× magnification.

### *In vivo* tumor xenograft study

All procedures for animal experiments were approved by the Committee on the Use and Care on Animals (Chongqing Medical University, Chongqing, China), and performed in accordance with the institution guidelines. Ovarian cancer SKOV3 or A2780 cells were infected with indicated lentiviral vectors and injected (5×10^6^ cells per mouse in 200 ul) subcutaneously into the left armpit of 6-week-old BALB/c nude mice. 21 days later, animals were sacrificed to confirm the presence of tumors and weigh the established tumors.

### Statistical analysis

All statistical analyses were performed using SPSS software, version 17.0 (Chicago, IL). Each experiment was performed in triplicates. Statistical analysis was performed by Student's t-test or analysis of variance (ANOVA). The chi-square test was used to compare the associations between MARCH7 overexpression and clinicopathologic variables of EOC samples. Data were presented as Mean ± standard deviation. Statistical significance was defined as a p-value less than 0.05.

## SUPPLEMENTARY MATERIAL, FIGURES


