# Supplementary material for: Ubiquitin E3 ligase MARCH7 promotes ovarian tumor growth
Source: Oncotarget. 2015 Mar 26;6(14):12174–87. doi: 10.18632/oncotarget.3650 (PMC4494930; doi:10.18632/oncotarget.3650)
Supplement: Supplementary file 1 [file oncotarget-06-12174-s001.pdf]

Ubiquitin E3 ligase MARCH7 promotes ovarian tumor growth

Supplementary Material

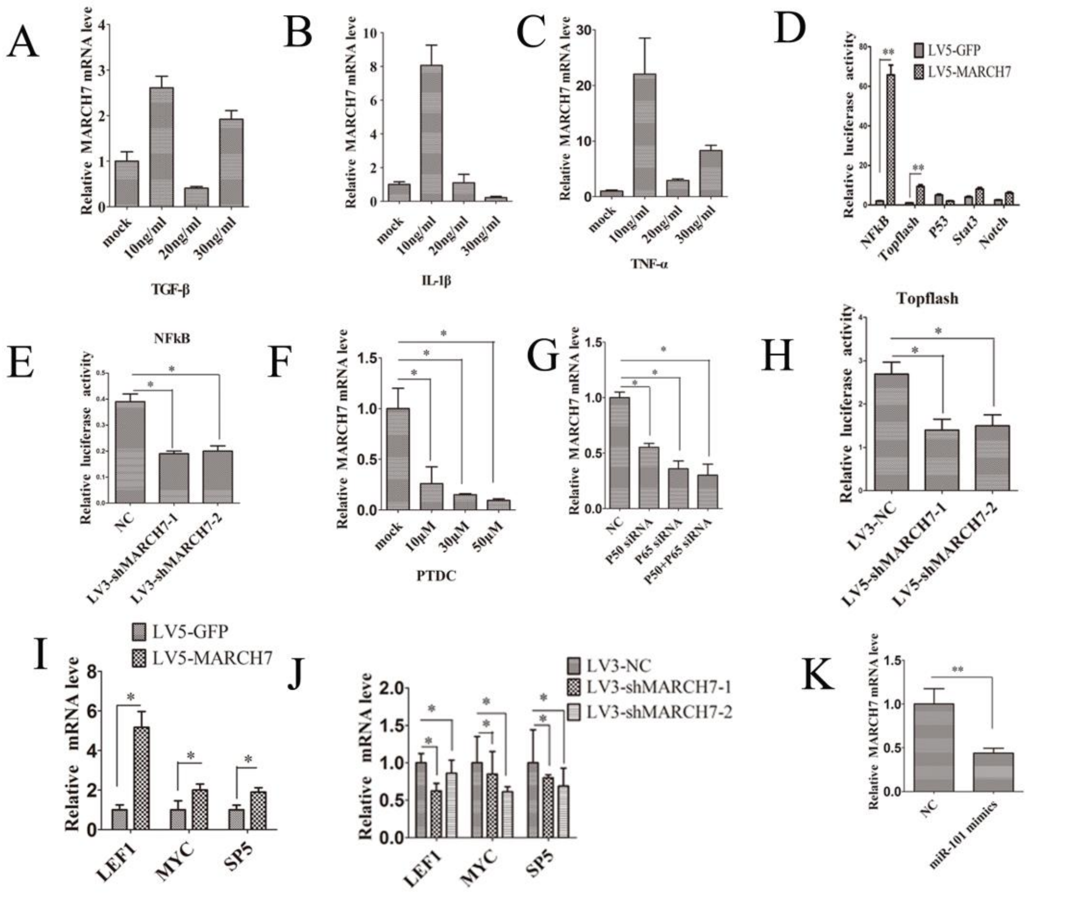

Figure S1: (A-C) The expression of MARCH7 mRNA level in SKOV3 cells was regulated by TGF-β1, TNF-α and IL-1β. (D) NF-κB, Notch, P53, STAT3 and Wnt/β-catenin signal pathway luciferase reporter activity was detected in ectopically expressed MARCH7 in ovarian cancer A2780 cells infected with LV5-MARCH7. (E) NF-κB signal pathway luciferase reporter activity was detected in LV3-shMARCH7-1, LV3-shMARCH7-2 and LV3-NC infected SKOV3 cells. (F, G) The expression of MARCH7

mRNA level in SKOV3 cells was regulated by PTDC, NFkB P50 and NFkB P65. (H) Wnt/β-catenin signal pathway luciferase reporter activity was detected in LV3-shMARCH7-1, LV3-shMARCH7-2 and LV3-NC infected SKOV3 cells. (I-K) The relative expression of mRNA level was detected by qPCR. Data are expressed as Mean ± SD from three independent experiments. * $p<0.05$, and ** $p<0.001$

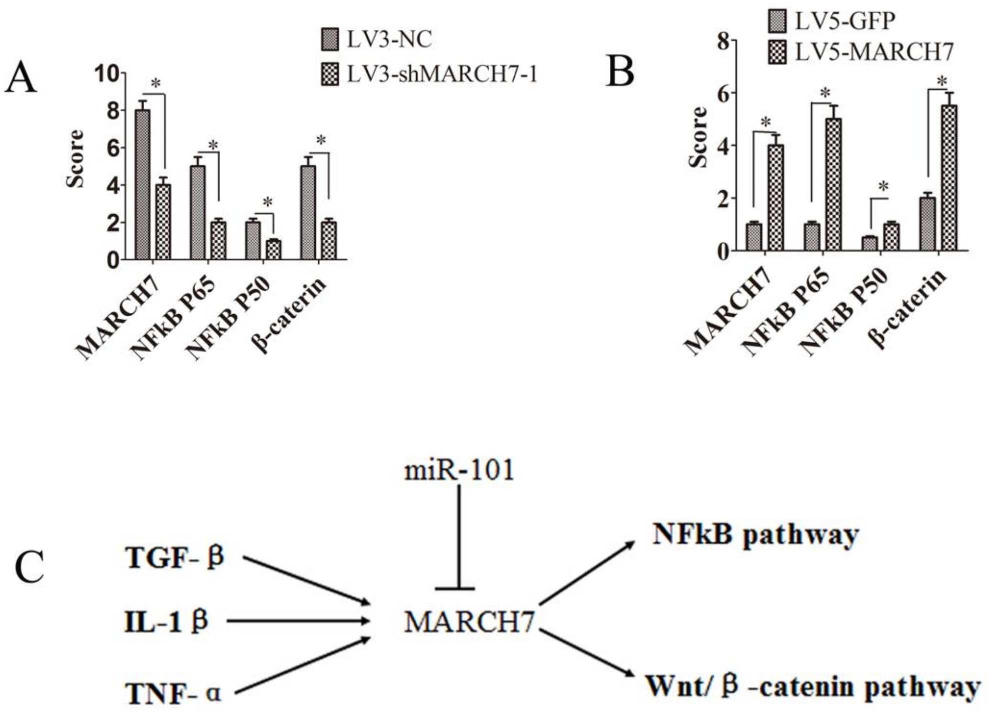

Figure S2: (A, B) Immunohistochemical score of MARCH7, NFkB P65, NFkB P50, and β-catenin expression were performed on tumor xenografts. (C) The graphic abstract for the significance of MARCH7 pathway (miR-101, TGF-beta1, TNF-alpha, IL-1beta, MARCH7, NFkB and Wnt/beta-catenin). Data are expressed as Mean ± SD from three independent experiments. * $p<0.05$, and ** $p<0.001$
